# Melatonin Blocks Morphine-Induced Place Preference: Involvement of GLT-1, NF-κB, BDNF, and CREB in the Nucleus Accumbens

**DOI:** 10.3389/fnbeh.2021.762297

**Published:** 2021-10-14

**Authors:** Badrah S. Alghamdi, Fahad S. Alshehri

**Affiliations:** ^1^Department of Physiology, Neuroscience Unit, Faculty of Medicine, King Abdulaziz University, Jeddah, Saudi Arabia; ^2^Pre-Clinical Research Unit, King Fahd Medical Research Center, King Abdulaziz University, Jeddah, Saudi Arabia; ^3^Department of Pharmacology and Toxicology, College of Pharmacy, Umm Al-Qura University, Makkah, Saudi Arabia

**Keywords:** morphine, addiction, melatonin, GLT-1, BDNF, NF-κB, CREB

## Abstract

Opioid addiction remains a widespread issue despite continuous attempts by the FDA to help maintain abstinence. Melatonin is a neurohormone considered to be involved only in the neuroendocrine and reproductive systems; however, recent reports have demonstrated its potential to attenuate drug addiction and dependence. Cumulative studies have suggested that melatonin can attenuate the rewarding effects of several drugs of abuse, including opioids. This study aimed to investigate the effect of melatonin (50 mg/kg) on morphine (5 mg/kg) to produce place preference. We also investigated the effect of melatonin and morphine on the expression of GLT-1, BDNF, NF-κB, and CREB within the nucleus accumbens. Male Wistar rats were divided into control, morphine, melatonin, and the morphine + melatonin groups. The study involved a two-phase habituation phase from day 1 to day 3 and an acquisition phase from day 5 to day 14. The conditioned place preference (CPP) score, distance traveled, resting time, ambulatory count, and total activity count were measured for all animals. Rats that received morphine showed a significant increase in CPP score compared to those in the control group. Morphine treatment reduced the mRNA expression of GLT-1, BDNF, and CREB and increased that of NF-κB. However, melatonin treatment administered 30 min before morphine treatment attenuated morphine place preference and reversed GLT-1, BDNF, NF-κB, and CREB expression levels. In conclusion, the study results indicate, for the first time, the new potential targets of melatonin in modulating morphine-induced CPP.

## Introduction

Opioid addiction is a complex phenomenon that heavily impacts economics, health care, and society as a whole (Motaghinejad et al., 2014). Morphine is commonly used in pain management and is considered the gold standard for opioids (Polomano et al., 2008; Jia et al., 2017; Mahshidfar et al., 2017). However, repeated use of morphine has been associated with significant changes in brain function and neural changes, especially in cases of long-term use (Allan et al., 2001; Spiga et al., 2005; Ballantyne, 2018). Several studies have suggested that long-term use of morphine is associated with depression, anxiety, and decline in cognitive function (Compton et al., 2000; Swendsen and Merikangas, 2000; Curran et al., 2001). Its long-term use is also associated with tolerance to its analgesic effect and withdrawal symptoms upon cessation (Mao et al., 2002; Desjardins et al., 2008). These side effects cause users to increase morphine doses, making cessation of its use difficult, as they become physiologically and psychologically dependent on the drug. Thus, there is an urgent need to investigate the molecular effects of morphine addiction and explore potential targets and possible treatments.

Cumulative evidence has established that the glutamatergic system plays an essential role in developing tolerance to morphine and the induction of addiction (Mao et al., 2002; Hao et al., 2005; Haghparast et al., 2007). Repeated administration of morphine can increase extracellular glutamate levels and potentiate glutamate excitotoxicity (Sepulveda et al., 2004; Jacobs et al., 2005). Therefore, several studies have linked the increase in extracellular glutamate levels in several brain areas such as the nucleus accumbens (NAc) with morphine-seeking behavior and withdrawal symptoms (Baharlouei et al., 2015; Yuan et al., 2017; Kim et al., 2018). Furthermore, repeated administration of morphine is associated with a reduction in glutamate clearance and downregulation of the glutamate transporter (Ozawa et al., 2001; Lim et al., 2005). The clearance of extracellular glutamate is mediated through several receptors such as glutamate receptor 1 (GLT-1) and cysteine-glutamate exchanger (Grewer et al., 2014). GLT-1 is considered the major transporter for glutamate clearance in the synaptic cleft, with approximately 90% of the total glutamate being transported into the glia through GLT-1 (Danbolt, 2001). Thus, glutamatergic excitotoxicity and glutamate clearance dysfunction are important therapeutic targets for morphine addiction.

Opioids can alter the immune system and induce neuroinflammation with prolonged morphine use (Roy and Loh, 1996; Sacerdote, 2006). The induced inflammatory responses can facilitate the mediation of neuroinflammatory cytokines and participate in activating transcription factor nuclear factor-kappa B (NF-κB; Roy et al., 1998; Hao et al., 2011; Nennig and Schank, 2017). Furthermore, NF-κB activates other transcription genes, regulates inflammation, and modulates synaptic processes, neurotransmission, and neuroprotection (Kaltschmidt and Kaltschmidt, 2009; Liu et al., 2017). It has been found that chronic morphine can increase NF-κB function *in vitro* (Sawaya et al., 2009) and elevated NF-κB expression in NAc (Hemby, 2004; Zhang et al., 2011). Therefore, blocking NF-κB in the NAc inhibited morphine-induced conditioned place preference (CPP) in rats (Zhang et al., 2011). Furthermore, morphine can modulate the cAMP response element-binding protein (CREB) transcription, which is involved in many neuronal processes, including neuronal survival, long-term memory, and morphine addiction (Yin and Tully, 1996; Walton and Dragunow, 2000; Martin et al., 2009). Also, it has been found that morphine administration is associated with reduced CREB levels in the NAc (Moron et al., 2010; Tenayuca and Nazarian, 2012), suggesting that behavioral adaptation in response to morphine-associated environmental cues is connected to the CREB signaling pathway. Also, several studies have shown that CREB is involved in morphine addiction (Yin and Tully, 1996; Walton and Dragunow, 2000; Martin et al., 2009). In fact, it has been reported that morphine-induced CPP was associated with lower expression levels of CREB in the NAc (Zhou and Zhu, 2006; Chen et al., 2012) and ventral tegmental area (Rezai et al., 2018). Furthermore, many studies have also described the effects of morphine addiction in the induction of neuroinflammatory regulators, including brain-derived neurotrophic factor (BDNF; Shen et al., 2012; Charkhpour et al., 2015). These changes were observed in morphine-treated animals; these animals showed lower expression levels of BDNF after serious escalating morphine doses (Fatahi et al., 2020). Therefore, the events of neuroinflammation and exploring these genes in the NAc could be a potential target in understanding morphine addiction.

The pineal gland secretes an endogenous indoleamine called melatonin (N-acetyl-5-methoxy-tryptamine; Hardeland et al., 2006; Ma et al., 2020). Melatonin is a neurohormone that activates the melatonin receptors (MTR1 and MTR2), modulating circadian rhythms and many physiological functions in mammals (Hardeland et al., 2006). Melatonin has long been considered to be involved only in the neuroendocrine and reproductive systems; however, several recent reports have suggested that melatonin activity exceeds that of a hormonal modulator. Melatonin has several physiological functions, including effects on mood, sleep, and immunomodulation, as well as antioxidant, and anti-inflammatory effects (Pandi-Perumal et al., 2008; Claustrat and Leston, 2015). In addition, the anti-inflammatory effect of melatonin has been shown to result in reduced NF-κB production, which in turn reduces the activity of several pro-inflammatory cytokines and inflammatory mediators (Beni et al., 2004; Li et al., 2005). Many studies have also claimed that melatonin has a strong neuroprotective effect against glutamate-induced excitotoxicity (Espinar et al., 2000; Lima et al., 2003; Vishnoi et al., 2016). Recently, melatonin has been shown to attenuate the rewarding behavior associated with many drugs of abuse such as cocaine (Sircar, 2000; Barbosa-Mendez and Salazar-Juarez, 2020) and alcohol (Vengeliene et al., 2015).

Cumulative studies have suggested that alcohol addiction is associated with reduced melatonin levels during sleep in humans and rodents (Peres et al., 2011; Crespi, 2012; Vengeliene et al., 2015). In addition, melatonin has been found to modulate the rewarding effects of many drugs of abuse, indicating that it plays an essential role in drug addiction (Onaolapo and Onaolapo, 2018). For example, it has been reported that melatonin attenuates cocaine-induced place preference and decreases both dopamine levels and locomotor sensitization in rats, whereas using luzindole (an MTR blocker) reverses the effects of melatonin (Barbosa-Mendez et al., 2021). Another study has shown that melatonin treatment can block cocaine self-administration and decrease relapse-like behavior (Takahashi et al., 2017). In addition, it has been reported that MTR1 and MTR2 knockout mice do not show place preference for methamphetamine, which further confirms the role of the melatonergic system in drug reward and addiction (Clough et al., 2014).

The NAc is an important brain region known to be critically involved in the learning process and rewards (Day and Carelli, 2007; Gold et al., 2019; Soares-Cunha et al., 2020). The NAc has different projections from and to multiple brain areas, including the prefrontal cortex (PFC), subcortical structures, hippocampus, amygdala, and ventral tegmental area (French and Totterdell, 2002; Xia et al., 2011, 2020; Piantadosi et al., 2017). Therefore, several studies have evaluated the contribution of the NAc in drug-related behaviors. For instance, opioid reinforcing and seeking effects are mediated through the dopaminergic and glutamatergic neurotransmission in the NAc (LaLumiere and Kalivas, 2008; Alshehri et al., 2018; Corre et al., 2018). Moreover, it has been found that morphine injection in the ventral tegmental area showed an augmented increase of the dopamine level in NAc (Leone et al., 1991). Recently, studies have shown that accumbal glutamate homeostasis is a potential target in attenuating cocaine-seeking in both humans (Engeli et al., 2020) and animals (Zhang et al., 2021).

Given the background mentioned above, this study investigated the effect of melatonin on morphine-induced CPP and the effect of melatonin on modulating morphine-induced changes in the expression of GLT-1, BDNF, NF-κB, and CREB in NAc. Thus, we show that the melatonin administration has effects on decreasing morphine-induced CPP and reversing some of the key markers of inflammation in the NAc.

## Materials and Methods

Male Wistar rats (weight 250–280 *g*) were obtained from the King Fahd Medical Research Center, King Abdulaziz University, Jeddah, and housed in plastic cages with free access to standard feed and water. Rearing conditions for the animals were as follows: 21°C, humidity 50%, and 12/12 light/dark cycle. The study was approved by the Animal Care and Use Committee (ACUC) guidelines of the King Fahd Medical Research Center. In addition, the experiments were approved by the Biomedical Ethics Research Committee (Reference 405-20) at King Abdulaziz University, following the guidelines of ethics and research on living creatures, prepared by the King Abdulaziz City for Science and Technology (KACST), approved by Royal Decree No. M/59 on 24 August 2010. Melatonin was purchased from Sigma-Aldrich (M5250); a fresh stock solution was prepared every day in 0.5% ethanol and diluted with saline (vehicle). Morphine was supplied by King Abdulaziz University Hospital Pharmacy (50 mg/kg, i.p.; Takahashi et al., 2017). The vehicle was composed of 0.5% ethanol and saline.

### Apparatus

The CPP apparatus consisted of two chambers made of Plexiglas and one small external chamber in the middle of the two chambers. The apparatus was obtained from Columbus Instruments, Columbus, OH, USA. The white chamber had vertical white stripes with a smooth white floor, whereas the black chamber had a small square of white and black and a small circle drilled in the floor. The apparatus was equipped with infrared sensors to measure the movement and activity of the animals during the test. In addition, an Auto-Track software (OPTO-MAX) was connected to the apparatus to automatically calculate time spent, ambulatory and distant movement, and resting time.

### Study Design and Animal Grouping

**Figure 1:**
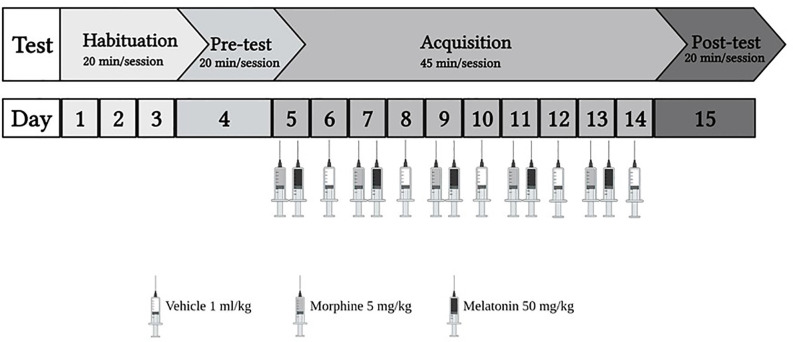
The experiment schedule for the habitation and acquisition of conditioned place preference (CPP). Animals underwent 3 days of habitation (20 min each day), followed by pre-test on day 4 (20 min). During the acquisition phase, the control group received vehicle (1 ml/kg), the morphine group received (5 mg/kg), the melatonin group received (50 mg/kg), and the morphine + melatonin received melatonin (50 mg/kg) 30 min before morphine (5 mg/kg) every other day alternatively between the drug paired chamber and the vehicle paired chamber (45 min each day). Then, at the end of the acquisition phase, which continued for 10 days, the post-test was conducted on day 15 (20 min). Morphine 5 mg/kg (i.p), melatonin 50 mg/kg.

The study consisted of two phases (Figure 1): the habituation phase, wherein from day 1 to day 3, animals explored the apparatus for 20 min with the partition divider raised between the two chambers. The habituation was done by placing the animal in the external chamber and then raising the gate to allow the animal to enter the apparatus. Then, on day 4, the pre-test was conducted for 20 min. The test was conducted by placing the animal in the external chamber and raising the gate to allow the animal to enter the apparatus. Once the animal entered the apparatus, the Auto-Track software (OPTO-MAX) automatically calculated the time spent, total activity count, ambulatory count, resting time, and distance traveled in each chamber. Most animals showed a preference for the black chamber. Therefore, a biased approach was used to avoid excluding many animals. Two animals were excluded due to diarrhea. Next, in the acquisition phase from day 5 to day 14, the partition divider separated the two chambers. Then, the post-test was conducted for 20 min on day 15, in which animals were placed in the apparatus with the partition divider raised, and the CPP score was calculated. On day 16, all the animals were euthanized using isoflurane.

Animals were divided into four groups (*n* = 7–8) as shown in Table 1. Control group, morphine group, melatonin group, and the morphine + melatonin group. The control group was administered the vehicle during the acquisition phase in the white and black chambers alternately for a total of 10 days. The morphine group received morphine i.p. (5 mg/kg) during the acquisition phase in the white chamber (drug-paired chamber) and the vehicle in the black chamber alternately for a total of 10 days. The melatonin group received melatonin i.p. (50 mg/kg) in the white chamber (drug-paired chamber) and the vehicle in the black chamber alternately during the acquisition phase for a total of 10 days. The morphine + melatonin group received melatonin i.p. (50 mg/kg) 30 min before morphine i.p. (5 mg/kg) in the white chamber (drug-paired chamber) and the vehicle in the black chamber alternately during the acquisition phase for a total of 10 days.

**Table 1:** Animals’ groups and treatment.

Animal groups	Treatment during acquisition
Control	Vehicle
Morphine	Morphine (5 mg/kg)
Melatonin	Melatonin (50 mg/kg)
Morphine + Melatonin	Melotonin (50 mg/kg) 30 min before Morphine (5 mg/kg)

*Control group (*n* = 7), morphine group (*n* = 8), melatonin (*n* = 8), morphine + melatonin (*n* = 7)*.

### Brain Tissue Collection

Animal brains were collected on day 16, immediately frozen, and stored at −80°C. The NAc was collected as pooled tissue containing NAc (core and shell) and isolated using a cryostat machine (Leica Biosystems). The NAc region (1.2–3.7 mm from bregma) was identified using the Brain Rat Atlas (Paxinos and Watson, 2006) and as performed previously in Alshehri et al. (2018) and Hammad et al. (2017).

### Real-Time Quantitative PCR

Total RNA was extracted from the NAc using the RNeasy Mini Kit (Qiagen, USA). Complementary DNA strand synthesis was performed using the cDNA synthesis kit (Sigma-Aldrich, UK). The qPCR quantification was done using the relative quantification approach by comparing the Ct value of (GLT-1, NFκB, CREB, and BDNF) to GAPDH, which was used as the reference gene for all mRNA expression analyses. All samples were run as triplicate, and the mean of the Ct value was taken. The relative expression of the tested genes was performed using the 2^−ΔΔCT^ method (Livak and Schmittgen, 2001; Deng et al., 2012). The Real-time qPCR was performed using the following primers to detect GLT-1- forward primer: 5′-GAGCATTGGTGCAGCCAGTATT-3′, reverse primer: 5′-GTTCTCATTCTATCCAGCAGCCAG-3′, NF-κB forward primer: 5′-GTCATCAGGAAGAGGTTTGGCT-3′, reverse primer: 5′-TGATAAGCTTAGCCCTTGCAGC-3′, BDNF forward primer: 5′-TCTACGAGACCAAGTGTAATCC-3′, reverse primer: 5′-TATGAACCGCCAGCCAAT-3′, CREB forward primer: 5′-CCAAACTAGCAGTGGGCAGT-3′, reverse primer: 5′- GAATGGTAGTACCCGGCTGA-3′, GAPDH (house-keeping gene) forward primer: 5′-CCCCCAATGTATCCGTTGTG-3′, reverse primer: 5′-TAGCCCAGGATGCCCTTTAGT-3′.

### Statistical Analysis

One-way analysis of variance (ANOVA) followed by Tukey’s *post hoc* tests was used to analyze the CPP score, distance traveled, resting time, ambulatory count, and total activity count. The CPP score was calculated as follows: (non-preferred chamber)/(total time spent in both chambers; Sun et al., 2018). In addition, one-way ANOVA was used to analyze the mRNA expression of GLT-1, NFκB, CREB, and BDNF. GAPDH was used as the reference gene for all mRNA expression analyses. All data were analyzed using Prism version 9.1.0. (*p*-value < 0.05).

## Results

### Effect of Melatonin on Morphine-Induced CPP

**Figure 2:**
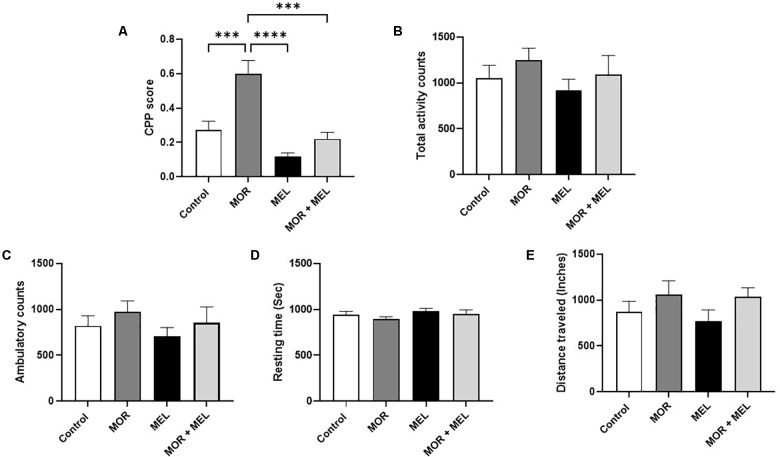
Effect of melatonin and morphine on CPP score **(A)**. The morphine group showed a higher CPP score compared to the control group and the melatonin group. Melatonin treatment 30 min before morphine (morphine + melatonin group) significantly lowers the CPP score than the morphine alone group. The melatonin group did not show any change in CPP score compared to the control group or the melatonin + morphine group. No changes were observed in total activity count **(B)**, ambulatory count **(C)**, resting time **(D)**, and distance traveled **(E)**. ***P < 0.001; ****P < 0.0001. MOR, morphine; MEL, melatonin.

The effect of melatonin on morphine-induced CPP was measured using the CPP paradigm. The analysis revealed a significant difference between the treatment groups (*F*_(3,26)_ = 17.10, *p* < 0.0001, Figure 2A). Further analysis with a Tukey’s *post hoc* test showed that animals that received morphine during the acquisition phase (morphine group) spent significant time in the drug-paired chamber with a higher CPP score compared to the control group (*p* = 0.0008) and the melatonin group (*p* < 0.0001). However, repeated melatonin treatment 30 min before receiving morphine (morphine + melatonin group) attenuated morphine-induced CPP, resulting in a significantly lower amount of time spent in the drug-paired chamber, with a lower CPP score than the morphine alone group (*p* = 0.0001). The melatonin-treated group did not show any significant change in CPP score compared to the control group (*p* = 0.1757) or the melatonin + morphine group (*p* = 0.5120).

### Effects of Melatonin and Morphine on Total Activity Count, Ambulatory Count, Resting Time, and Distance Traveled

Analysis of the total activity count, which calculates any beam break due to animal movement, including stereotypical movement (scratching or grooming behavior), showed that the total travel distance did not differ significantly between the treatment groups (*F*_(3,24)_ = 0.7659, *p* = 0.5244, Figure 2B). In addition, the ambulatory count, which is the number of instances when the animal breaks the beams inside both chambers, excluding instances of stereotypic movement, did not differ significantly between the treatment groups (*F*_(3,24)_ = 0.7460, *p* = 0.5353, Figure 2C). Moreover, the resting time, representing the time when the animal did not move inside the chambers, did not change significantly between the treatment groups (*F*_(3,27)_ = 1.336, *p* = 0.2833, Figure 2D). The total distance traveled inside the chamber, calculated in inches, did not significantly differ between the treatment groups (*F*_(3,23)_ = 1.280, *p* = 0.3048, Figure 2E).

### Effect of Melatonin and Morphine on GLT-1 mRNA Expression

**Figure 3:**
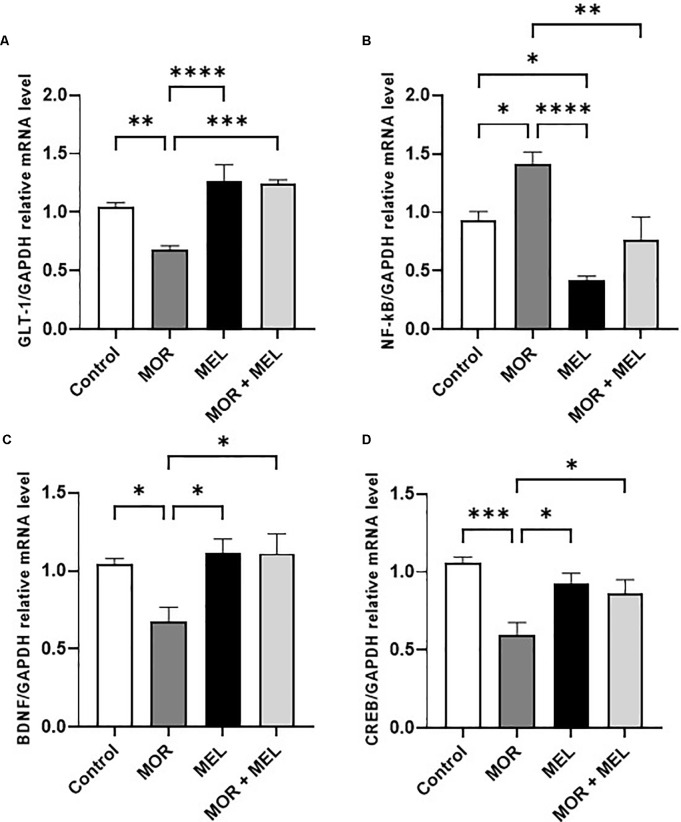
Effect of melatonin and morphine on GLT-1 mRNA expression **(A)**. Morphine treatment decreased GLT-1 mRNA expression compared to the control and melatonin group; however, melatonin treatment in (morphine + melatonin) restored GLT-1 mRNA expression. Effect of melatonin and morphine on NF-κB mRNA expression **(B)**. Morphine treatment decreased NF-κB mRNA expression compared to the control and melatonin group; however, melatonin treatment in (morphine + melatonin) reduced NF-κB mRNA expression. Effect of melatonin and morphine on BDNF **(C)** and CREB **(D)** mRNA expression. Morphine treatment decreased BDNF and CREB mRNA expression compared to the control and melatonin group; however, melatonin treatment in (morphine + melatonin) restored BDNF and CREB mRNA expression (*P < 0.05; **P < 0.01; ***P < 0.001; ****P < 0.0001). MOR, morphine; MEL, melatonin; GLT-1, glutamate transporter-1; CREB, cAMP response element-binding protein; BDNF, brain-derived neurotrophic factor.

The effect of melatonin and morphine on GLT-1 mRNA expression was also measured. Significant difference was found between the treatment groups (*F*_(3,24)_ = 13.23, *p* < 0.0001, Figure 3A). Additional analysis with Tukey’s *post hoc* test showed that animals that received morphine during the acquisition phase (morphine group) had lower GLT-1 expression compared to those in the control group animals (*p* = 0.0093). However, repeated melatonin treatment 30 min before morphine (morphine + melatonin group), which attenuated morphine-induced CPP, successfully prevented the reduction in GLT-1 expression (*p* = 0.0001), with no significant difference in GLT-1 levels compared to the control group (*p* = 0.2914).

### Effect of Melatonin and Morphine on NF-κB, BDNF, and CREB mRNA Expression

The effect of melatonin and morphine on NF-κB mRNA expression was also analyzed. The results showed a significant difference between the treatment groups (*F*_(3,24)_ = 12.48, *p* < 0.0001, Figure 3B) and that animals that received morphine during the acquisition phase (morphine group) had a higher NF-κB expression level compared to the control group (*p* = 0.0363). However, repeated melatonin treatment 30 min before morphine (morphine + melatonin group) was associated with blocking morphine-induced CPP and reduction in the expression of NF-κB compared to that in the morphine group (*p* = 0.0032), and restored NF-κB expression compared to that in the control group (*p* = 0.7331). Moreover, repeated treatment with melatonin alone (melatonin group) significantly reduced NF-κB expression compared to that in the control group (*p* = 0.0239).

With respect to the mRNA expression of BDNF, a significant difference between the treatment groups was found (*F*_(3,24)_ = 5.145, *p* = 0.0069, Figure 3C). The results showed that animals in the morphine group had a lower BDNF expression level than those in the control group (*p* = 0.0425). However, repeated melatonin treatment 30 min before morphine (morphine + melatonin group) increased the expression of BDNF compared to that in the morphine group (*p* = 0.0136) and reversed BDNF expression compared to that in the control group (*p* = 0.9576). Repeated treatment with melatonin alone (melatonin group) did not induce any change in BDNF expression compared to that in the control group (*p* = 0.9560).

Analysis of the mRNA expression of CREB showed a significant difference between the treatment groups (*F*_(3,24)_ = 8.080, *p* = 0.0007, Figure 3D). Further analysis showed that the morphine group had a lower CREB expression level compared to the control group (*p* = 0.0004). However, repeated melatonin treatment 30 min before morphine (morphine + melatonin group) increased the expression of CREB compared to that in the morphine group (*p* = 0.0499) and did not affect the CREB expression compared to that in the control group (*p* = 0.2082). Repeated treatment with melatonin alone (melatonin group) did not induce any change in CREB expression compared to that in the control group (*p* = 0.5276).

## Discussion

The CPP paradigm is a vital tool used to measure different drug rewards based on Pavlov’s conditioning principles (Bardo and Bevins, 2000; Tzschentke, 2007). Therefore, drugs of abuse, such as opioids, act as conditional stimuli and produce reinforcing effects in humans and rodents (Shen et al., 2017; Alshehri et al., 2018; Zhu et al., 2019). In the CPP paradigm, animals receive an appetitive stimulus (such as morphine) and associate the reward with contexts and cues in the drug-paired chamber inside the CPP apparatus (Kobrin et al., 2016; Meepong and Sooksawate, 2019). Thus, an increase in the time spent in the drug-paired chamber than in the control chamber during the post-test is considered to support the drug’s reinforcing effect (Kobrin et al., 2016; Meepong and Sooksawate, 2019). In this study, we aimed to investigate the effect of melatonin on morphine-induced CPP using the CPP paradigm. Morphine conditioning was established in these animals by repeated administration of morphine in a drug-paired chamber over five sessions. However, melatonin treatment before morphine administration successfully attenuated morphine-induced CPP during the post-test.

The dopaminergic system is involved in drug abuse, reinforcing effects, and addiction (Solinas et al., 2019). It is known that homeostasis between the dopaminergic and DA and glutamatergic is essential in regulating cortico-striatal rhythms, where its imbalance can enhance cognitive dysfunctions and motor deficits (Bamford et al., 2004; Calabresi et al., 2007; Gleich et al., 2015). Repeated use of drugs of abuse could impair the capacity of PFC input to the NAc to regulate drug-seeking behavior in corticostriatal circuitry (Scofield et al., 2016). Cumulative studies have extensively investigated the role of dopaminergic neurotransmission in the rewarding properties of morphine by examining genetic alteration or deletion of dopamine receptors (Smith et al., 2002; Francès et al., 2004) and through using dopamine receptor modulator (Manzanedo et al., 2001; Frances et al., 2004; Assar et al., 2016) in the CPP paradigms. Moreover, several reports have suggested that the glutamatergic system also participates in drug reward and seeking behavior (Kalivas, 2004; Kalivas et al., 2005). Deficiency in glutamate homeostasis leads to drug-induced synaptic plasticity and morphological changes in the dendritic spines in the NAc area (Robinson and Kolb, 1997). Thus, using transferring the GLT-1 gene into the NAc by using recombinant adenoviruses techniques, has attenuated morphine-induced CPP (Fujio et al., 2005). In addition, injecting glutamate receptor antagonists such as NMDA blocker in the NAc was revealed to attenuate morphine-induced CPP in rats (Bespalov and Zvartau, 1996). Altering glutamate homeostasis has also been reported with other abuse drugs such as cocaine (Kalivas, 2004), alcohol (Dodd et al., 2000), nicotine (Lambe et al., 2003), cannabinoids (Brown et al., 2003), and opioids (Fundytus, 2001). Thus, restoring glutamate homeostasis is a potential target for drug reward and addiction. In this study, morphine-induced place preference was associated with a reduction in GLT-1 mRNA expression. Melatonin treatment before morphine reversed GLT-1 expression levels in the NAc. Therefore, the pattern of GLT-1 alteration is similar to that observed in other models of addiction, and thus GLT-1 could be an appropriate target for future functional examination using microdialysis or pharmacological techniques.

Moreover, this study evaluated the effect of morphine-induced CPP on the expression of NF-κB, BDNF, and CREB in the NAc. As a result, morphine was associated with a higher mRNA expression level of NF-κB. Previously, drugs of abuse were known to activate NF-κB, indicating the functional role of NF-κB’s rewarding effects. For example, inhibiting NF-κB in the NAc was shown to attenuate morphine-induced place preference in rats (Zhang et al., 2011). For instance, fear memory inhibiting NF-κB was shown to reduce inhibitory avoidance and memory reconsolidation in mice (Freudenthal et al., 2005; Merlo and Romano, 2008). These studies implied that the NF-κB is involved in mediating morphine-CPP. On the other hand, we found that melatonin treatment reverses the upregulation of NF-κB mRNA expression associated with morphine treatment. Similarly, exploring melatonin effect on D-galactose-induced memory impairment, which was associated with the elevated expression level of NF-κB in the cortex, reverse D-galactose induced memory impairment and neuroinflammation (Ali et al., 2015). Therefore, the NF-κB may act as a contributory factor in the neuronal reward mediating the effects of morphine in CPP. Our results suggest that melatonin administration modulates morphine-induced CPP, which was associated with reversing NF-κB expression in NAc. This effect is more likely due to the anti-inflammatory properties of melatonin to reduce NF-κB and several pro-inflammatory cytokines and inflammatory mediators (Beni et al., 2004; Li et al., 2005).

On the other hand, in this study, morphine was associated with a lower mRNA expression level of CREB. Similarly, several reports have suggested morphine-induced preference in CPP was associated with lower expression levels of CREB in the NAc (Zhou and Zhu, 2006; Chen et al., 2012) and ventral tegmental area (Rezai et al., 2018). Furthermore, several studies have shown that CREB is involved in morphine addiction (Yin and Tully, 1996; Walton and Dragunow, 2000; Martin et al., 2009). In fact, it has been found that morphine is associated with reduced CREB levels in the NAc (Moron et al., 2010; Tenayuca and Nazarian, 2012), suggesting that morphine behavioral adaptation is connected to CREB. Also, several studies have reported that morphine can suppress BDNF signaling in the NAc, which was associated with reducing inhibitory GABAergic and enhanced morphine reward (Koo et al., 2014). Importantly, in this study, melatonin treatment reverses morphine associated lower CREB mRNA expression in NAc. Similarly, melatonin treatment was shown to provide a potent antioxidant and neuroprotectant effect against Polychlorinated Biphenyls (PCBs) treated rats as a model of neurotoxicity with lower CREB gene expression. Thus, melatonin treatment attenuated PCBs effect and elevated CREB gene expression cerebral cortex in these rats (Bavithra et al., 2015). Therefore, our results suggest that the modulatory effect of melatonin attenuating morphine-induced CPP could be partly due to melatonin inducing cellular changes in CREB expression level in the NAc.

This study examined the effect of morphine on lowering mRNA expression level of BDNF. It has been reported that repeated morphine administration can lower BDNF expression in the ventral tegmental area (Koo et al., 2012). A similar finding was also reported by Rezai et al. (2018) after repeated morphine treatment was associated with lower BDNF levels in the ventral tegmental area. Consequently, resulting in attenuating BDNF signaling from the ventral tegmental area to the NAc and participating in morphine rewarding circulatory. In addition, it has been reported that TrκB receptor antagonist’s (BDNF receptor antagonist) systemic injection facilitates morphine dependency and withdrawal effects in rats (Rezamohammadi et al., 2020). In our study, we reported that melatonin treatment reversed morphine associated lower BDNF mRNA expression in NAc. In fact, it has been reported that melatonin treatment attenuated methamphetamine-induced downregulation of BDNF expression levels in mice (Veschsanit et al., 2021). Thus, the ability of melatonin treatment to attenuate morphine-induced CPP could be described through modulating these targets in the NAc.

Moreover, other behavioral parameters such as distance traveled, resting time, ambulatory count, and total activity count were evaluated to provide a more precise idea of animal behavior during the post-test. The results showed that morphine-induced CPP and melatonin treatment did not significantly affect these parameters in the post-test. This is in agreement with previous studies showing that the morphine-induced CPP does not affect locomotion or distance traveled (Farzinpour et al., 2019). In fact, it is essential to understand that melatonin did not affect animal locomotion or induce any behavior that may have a confounding effect, such as sedation or an aversion effect. In fact, animals that received repeated doses of melatonin did not show any significant behavioral changes when evaluated for CPP score, distance traveled, resting time, ambulatory count, and total activity count.

This study was intended to investigate the effect of melatonin on morphine-induced CPP and the effect of melatonin and morphine on the expression of GLT-1, BDNF, NF-κB, and CREB within the NAc brain region. It is essential to note that the changes in the expression of these targets are not only limited to morphine-induced CPP. In fact, It has been suggested that sucrose withdrawal after long-term exposure is associated with the inactivation of CREB in NAc (Kim et al., 2018). Also, repeated variable stress was shown to enhance nicotine-seeking behavior and decreased CREB in the NAc (Leao et al., 2012). Also, cumulative studies have linked reduction of GLT-1 expression to several drugs reward and relpase such as methamphetamine (Siemsen et al., 2019), cocaine (Trantham-Davidson et al., 2012), and alcohol (Das et al., 2015). Thus, giving the GLT-1 in NAc a common target for multiple drugs of abuse. Future studies are warranted to investigate GLT-1, BDNF, NF-κB, and CREB and assess their function in natural reward of other compounds and their involvement in other brain areas.

## Conclusion

There is an increasing need to investigate and understand the molecular effects of morphine addiction and explore potential targets and possible treatments. Glutamatergic excitotoxicity, neuroinflammation, and glutamate clearance dysfunction are important therapeutic targets for morphine addiction. Thus, we investigated the effect of melatonin on morphine-induced place preference using the CPP paradigm. Melatonin treatment before morphine administration successfully attenuated morphine-induced CPP. This was associated with reversing the morphine-induced changes in GLT-1, NF-κB, BDNF, and CREB expression in the NAc brain region. This shows that melatonin blocking effect against morphine-induced CPP could be through modulating glutamate transporter, neurotrophins, and neuroinflammatory targets.

## Data Availability Statement

The original contributions presented in the study are included in the article, further inquiries can be directed to the corresponding author.

## Ethics Statement

The animal study was reviewed and approved by the Animal Care and Use Committee (ACUC) guidelines of the King Fahd Medical Research Center. In addition, the experiments were approved by the Biomedical Ethics Research Committee (Reference 405-20) at King Abdulaziz University, following the guidelines of ethics and research on living creatures, prepared by the King Abdulaziz City for Science and Technology (KACST), approved by Royal Decree No. M/59 on 24 August 2010.

## Author Contributions

BA and FA worked on conceptualization, methodology, validation, data analysis, investigation, writing, reviewing, and editing the study. All authors contributed to the article and approved the submitted version.

## Conflict of Interest

The authors declare that the research was conducted in the absence of any commercial or financial relationships that could be construed as a potential conflict of interest.

## Publisher’s Note

All claims expressed in this article are solely those of the authors and do not necessarily represent those of their affiliated organizations, or those of the publisher, the editors and the reviewers. Any product that may be evaluated in this article, or claim that may be made by its manufacturer, is not guaranteed or endorsed by the publisher.

## Abbreviations

NAc: nucleus accumbens
GLT-1: glutamate transporter-1
CREB: cAMP response element-binding protein
BDNF: brain-derived neurotrophic factor.
